# Detention of Dr. Binayak Sen: Something Must Be Seriously Wrong Somewhere

**DOI:** 10.4103/0970-0218.43224

**Published:** 2008-10

**Authors:** Sanjay Chaturvedi

**Affiliations:** Department of Community Medicine, University College of Medical Sciences and GTB Hospital, Delhi, India

For over a year, Dr. Binayak Sen, a medical professional who transcended the role of merely being a doctor, is languishing in jail. He was arrested by the Chhattisgarh police on 14 May 2007 and was sent to Raipur Central Jail under the Chattisgarh Special Public Security Act (CSPSA), 2005 and the Unlawful Activities Act (UAPA), 2004. The police allege, without proof, that he was passing notes from a Naxalite he was treating in jail, while civil society organisations and wider scientific fraternity, including 22 Nobel Laureates, assert that Dr. Sen appears to be incarcerated solely for exercising his fundamental right, and this detention is in contravention of Articles 19 (freedom of opinion and expression) and 22 (freedom of association) of the international covenant on civil and political rights—to which India is a state party.

Dr. Sen's bail petition was rejected by the high court on 23 July 2007 and by the Supreme Court on 12 December 2007. His application for parole to receive the Keithan gold medal awarded to him in December 2007 by the Indian Academy of Social Sciences was also rejected on technical grounds. He was kept in solitary confinement for nearly a month in March-April 2008 for no apparent reason. Police justified their action by claiming that Dr. Sen was kept in isolation for his own security but failed to explain the nature or source of the threat to him as a regular prisoner. His status as a regular prisoner was restored only after widespread protests. Amidst painful delays in legal proceedings, his trial began on 30 April 2008. It may be important to mention here that Dr. Sen is 58 years old, is suffering from cardiac disease, and has lost 20 kgs of body weight during incarceration. Till now, the police and administration has failed to get any hard evidence to incriminate Dr. Sen, but that hasn't stopped them from charging Dr. Sen with serious offences, enough to be punished with life imprisonment or even a death sentence. He has been accused of being a member of a terrorist gang or organisation, knowingly holding proceeds of terrorism, conspiracy to wage war against the state, and sedition.

For the marginalised people of Chhattisgarh, Dr. Sen needs no introduction. For those who don't know him, he did his M.D. in pediatrics from the Christian Medical College (CMC) in Vellore and worked for a couple of years as a teacher of public health at JNU, Delhi. Soon he returned to his priorities, shifted to central India, and focused on improving the health care and living conditions of the poorest of the poor in tribal regions. This lifetime work started in 1981 when he helped to establish the Shaheed Hospital at Dalli-Rajhara where the very people who built the hospital were able to use its facilities. This pioneering effort by workers to build and run their own hospital and healthcare facility has become a model for effective and low-cost care for the marginalised. In 1994, he and his wife set up an NGO, *Rupantar,* to provide medical and healthcare facilities for the poor. Dr. Sen also has a political profile to his activism. He became associated with the People's Union for Civil Liberties, an organisation initiated by Jaiprakash Narayan. In the local context, the organisation has been opposing the use of excessive and unwarranted police power in the name of resolving the Naxalite problem.

Any student or young aspirant of medicine who would learn about these stories would be caught in the cleft. On one hand, we have an accepted model of successful doctors, first acquiring basic medical qualifications at the cost of people of India and then migrating to serve distant establishments and societies distinctly designed to defeat developing economies. On the other hand, we have exceptional examples of individuals breaking these models of spurious and notorious success who end up inviting the wrath of local establishments erected in the name of the same people they are serving. On one hand, we are reducing our medical schools to higher education corporates producing cheap manpower for developed economies. On the other hand, we are lamenting that our doctors are not reaching marginalized people. There must be something seriously wrong somewhere.

The behavior of the state in the developing economies has been consistently disturbing. At the facade, they call for empowerment and community-owned actions but when the same positive designs start taking the shape of reality, they feel threatened. One can argue that people like Dr. Sen invite trouble because of their political activism and not because of their welfare oriented work. We need to examine this argument in light of the counterpoint that an issue like empowerment cannot be successfully addressed by totally apolitical actions. Sometimes, being apolitical can be a very handy logic for perpetually maintaining safe, meaningless yet profitable positions.

One can also argue that such issues should not have a space in an academic biomedical journal. Nevertheless, how long can academics remain apolitical when it is being increasingly felt that governance in health is going to be one of the most critical discourses in public health during the coming decades? If anything, medicalisation of public health has already blunted its political arguments. The issue is not that of being or not being political—it is of how to take a rational political stand on health while preserving the mandatory space of objective distance for scientific examination. Objective distance in itself is a contested territory. Science and technology have their own social and political constructs and are not necessarily value free.

